# Voltage Dependence of a Neuromodulator-Activated Ionic Current^1^,^2^,^3^

**DOI:** 10.1523/ENEURO.0038-16.2016

**Published:** 2016-05-12

**Authors:** Michael Gray, Jorge Golowasch

**Affiliations:** 1 Behavioral and Neural Science Graduate Program, Rutgers University-Newark, Newark, New Jersey 07102; 2 Federated Department of Biological Sciences, New Jersey Institute of Technology, Newark, New Jersey 07102

**Keywords:** activity, calcium sensing receptor, calcium-dependence, central pattern generation, crustacean, stomatogastric

## Abstract

The neuromodulatory inward current (*I*_MI_) generated by crab *Cancer borealis* stomatogastric ganglion neurons is an inward current whose voltage dependence has been shown to be crucial in the activation of oscillatory activity of the pyloric network of this system. It has been previously shown that *I*_MI_ loses its voltage dependence in conditions of low extracellular calcium, but that this effect appears to be regulated by intracellular calmodulin. Voltage dependence is only rarely regulated by intracellular signaling mechanisms. Here we address the hypothesis that the voltage dependence of *I*_MI_ is mediated by intracellular signaling pathways activated by extracellular calcium. We demonstrate that calmodulin inhibitors and a ryanodine antagonist can reduce *I*_MI_ voltage dependence in normal Ca^2+^, but that, in conditions of low Ca^2+^, calmodulin activators do not restore *I*_MI_ voltage dependence. Further, we show evidence that CaMKII alters *I*_MI_ voltage dependence. These results suggest that calmodulin is necessary but not sufficient for *I*_MI_ voltage dependence. We therefore hypothesize that the Ca^2+^/calmodulin requirement for *I*_MI_ voltage dependence is due to an active sensing of extracellular calcium by a GPCR family calcium-sensing receptor (CaSR) and that the reduction in *I*_MI_ voltage dependence by a calmodulin inhibitor is due to CaSR endocytosis. Supporting this, preincubation with an endocytosis inhibitor prevented W7 (*N*-(6-aminohexyl)-5-chloro-1-naphthalenesulfonamide hydrochloride)-induced loss of *I*_MI_ voltage dependence, and a CaSR antagonist reduced *I*_MI_ voltage dependence. Additionally, myosin light chain kinase, which is known to act downstream of the CaSR, seems to play a role in regulating *I*_MI_ voltage dependence. Finally, a Gβγ-subunit inhibitor also affects *I*_MI_ voltage dependence, in support of the hypothesis that this process is regulated by a G-protein-coupled CaSR.

## Significance Statement

Neurons and neuronal networks display many forms of activity, of which oscillatory activity is crucial in many vital functions such as heartbeat, digestion, and locomotion. The state in which a neuron exists is often determined by its neuromodulatory environment. Recent studies have shown that many neuromodulators that enable oscillatory activity in neurons do so by activating voltage-gated inward currents that express a negative slope conductance. Such voltage gating is normally an intrinsic property of the ion channels themselves and is only rarely mediated by a separate signaling pathway or molecule. Here we characterize what we believe is a novel voltage dependence mechanism that involves active extracellular calcium sensing by a dedicated receptor, and intracellular calcium-dependent processes that modulate the voltage dependence of this current.

## Introduction

Neuromodulators enhance the flexibility of neural networks by multiple mechanisms. These include the regulation of intrinsic properties (i.e., pre-existing conductances; Benson and Levitan, 1983; Kiehn and Harris-Warrick, 1992a,b; Harris-Warrick et al., 1995; Galbavy et al., 2013), modulating synaptic properties (Johnson et al., 1993a,b,1995; Zhao et al., 2011; Bitencourt et al., 2015; Böhm et al., 2015), reconfiguring participating neurons within the network (Hooper and Moulins, 1989; Fénelon et al., 1998; Lieske et al., 2000), and modulating plasticity and even modulation itself (Mesce, 2002; McLean and Sillar, 2004; Zhou et al., 2007; Pawlak et al., 2010; Lawrence et al., 2015). Another important mechanism of the regulation of neuronal excitability is the modulation of leak currents (Bayliss et al., 1992; Erxleben et al., 1995; Talley et al., 2000; Cymbalyuk et al., 2002; Xu et al., 2009), and of the ratio of leak current to pacemaking current amplitude (and not just the pacemaking current amplitude), as is the case in the regulation of pacemaking activity in the pre-Bötzinger complex (Del Negro et al., 2002). The regulation of inward currents whose current–voltage relationship (*I–V*) curve contains a region of negative slope (i.e., negative conductance) often plays a major role in the control of excitability and pacemaking activity (Nowak et al., 1984; Freschi, 1989; Trimmer, 1994; Haj-Dahmane and Andrade, 1996; Zholos and Bolton, 1996; Shiells and Falk, 2001; Xu et al., 2009; Zhao et al., 2010). Such a negative conductance region can be approximated well by an *I–V* curve with linear negative conductance (Zhao et al., 2010; Bose et al., 2014). When this region occurs in the voltage range of oscillatory activity, neuromodulatory regulation of these currents can be thought of as the regulation of leak currents (Haj-Dahmane and Andrade, 1996; Zhao et al., 2010; Bose et al., 2014). Recently, it was demonstrated that the negative conductance region of the *I–V* curve of any inward current acts to destabilize the neuronal resting state, thus pushing the membrane potential away from the resting voltage, without the need that the ionic current be nonlinear around the resting potential (Bose et al., 2014). The negative conductance of an *I–V* curve is normally generated by an interaction of voltage-dependent ion channel gating and a depolarized equilibrium potential. However, other mechanisms that produce negative conductance exist, such as the voltage-dependent magnesium blockade of the NMDA receptor (Nowak et al., 1984). Additionally, there are few examples that describe voltage-dependent mechanisms mediated by intracellular signaling pathways (Zholos and Bolton, 1996; Nawy, 2000; Shiells and Falk, 2001).

Here, we use the stomatogastric ganglion (STG) of the crab *Cancer borealis* to explore how the voltage dependence of a neuromodulator-activated current is controlled. This system enables unambiguous cell-type identification and is a practical system for studying negative slope conductance as a mechanism of neuromodulator-induced oscillatory activity (Zhao et al., 2010; Bose et al., 2014). Specifically, we examine the voltage dependence of the modulator-activated inward current (*I*_MI_). This current is thought to be the primary mechanism by which a set of peptide neuromodulators activate the oscillatory activity of the pyloric network (Swensen and Marder, 2000, 2001), thanks to its region of negative slope conductance (Zhao et al., 2010). *I*_MI_ was characterized originally as an inward current activated by proctolin, whose voltage dependence is sensitive to extracellular calcium, in the absence of which *I*_MI_ becomes linear (Golowasch and Marder, 1992b). The authors suggested a mechanism similar to that of the NMDA receptor (Nowak et al., 1984) to explain the nonlinear properties of the current: a voltage-dependent block of the channel by extracellular calcium (rather than magnesium). A later study (Swensen and Marder, 2000) found that when exposed to the calmodulin inhibitor *N*-(6-aminohexyl)-5-chloro-1-naphthalenesulfonamide hydrochloride (W7), *I*_MI_ amplitude was enhanced and its voltage dependence seemed to be altered, suggesting a modulation of the current by calmodulin. The main hypothesis that we address here is that the voltage dependence of *I*_MI_ is mediated by an extracellular activation of a calcium-sensing mechanism mediated by a calcium-dependent intracellular signaling pathway.

## Materials and Methods

### Animals

Male crabs of the species *C. borealis* were purchased from local fisheries, housed in saltwater aquaria at 8-12°C, and randomly picked. The animals were anesthetized on ice for at least 30 min prior to dissection. The stomatogastric nervous system (STNS) was dissected out and pinned on Sylgard dishes, as previously described (Maynard and Dando, 1974; Selverston et al., 1976). The isolated STNS was continuously perfused with chilled saline solution (12-14°C), which was composed of the following: 440 mm NaCl, 11 mm KCl, 13 mm CaCl_2_, 26 mm MgCl_2_, 5 mm maleic acid, and 11 mm Trizma base, and was adjusted to a pH of 7.4-7.5. For low-calcium solutions, MgCl_2_ was added in equimolar amounts to compensate for reduced calcium levels. In all experiments, STG neurons and neuropil were exposed by desheathing and pinning down the surrounding connective tissue. Unless otherwise noted, all data reported here were obtained from lateral pyloric (LP) neurons. Concentrations of calcium at or below a concentration of 2 mm were found to depolarize LP cells (mean resting potential, from −48.9 ± 2.1 to −34.2 ± 2.1 mV; *n* = 9; paired Student’s *t* test (*t*_(8)_ = −15.2, *p* = 3.6 × 10^−7^])^a^ and decrease their input resistance (mean measured at −50 mV; from 9.4 ± 1.8 to 5.4 ± 0.7 MΩ; *n* = 9; *t* test: *t*_(8)_ = 3.5, *p* = 0.008)^b^. To attenuate this effect, low-calcium saline solution was supplemented with 0.5% bovine serum albumin (BSA). This prevented low calcium-induced depolarization (mean resting potential in low-calcium condition: no BSA, −26.0 ± 2.1 mV (*n* = 5); low calcium level plus 0.5% BSA, −45.1 ± 2.4 (*n* = 7); Student’s *t* test: *t*_(10)_ = 5.8, *p* = 1.77 × 10^−4c^), but this treatment had no significant effect on input resistance (median input resistance in low-calcium condition: no BSA, 5.5 MΩ; low calcium level plus 0.5% BSA, 6.0 MΩ (*n* = 7); Mann–Whitney rank sum *U* test: *U* = 12, *p* = 0.413^d^). Therefore, all low-calcium experiments were supplemented with 0.5% BSA, except for R568 experiments. R568 was used to test the role of the calcium-sensing receptor (CaSR), but R568 is known be activated by amino acids (Conigrave et al., 2007) and albumin hydrolysates (Nakajima et al., 1962). Because the STG neuropil is known to contain active peptidases (Coleman et al., 1994), which may release these compounds, BSA was not used when R568 was tested.

### Electrophysiology

Extracellular recordings were made using Vaseline wells built around lateral ventricular or dorsal ventricular nerves, and with one stainless steel wire inserted in each well and one outside, both connected to an A-M Systems Model 1700 Differential AC Amplifier. Ground electrodes were either AgCl pellets (Molecular Devices) or chloride-coated silver wires (coating was obtained by inserting the silver wire in bleach for ≥15 min). All intracellular recordings, unless otherwise stated, were obtained with an Axoclamp 2B Amplifier (Molecular Devices) and digitized with a Digidata 1322A or 1440 Digitizer (Molecular Devices) and recorded onto a PC with a Microsoft Windows operating system using the pClamp 9 or 10.4 software suite (Molecular Devices). Currents were recorded in two-electrode voltage clamp (TEVC) and passively filtered using an RC filter at a 4 KHz cutoff frequency before digitization. Microelectrodes were pulled on a Sutter P-97 Puller with resistances of 15-25 MΩ for the voltage recording electrode (ME1) and 10-20 MΩ for the current passing electrode (ME2). All recording solutions consisted of 0.6 M K_2_SO_4_ plus 20 mm KCl.

### Neuromodulators

Proctolin and crustacean cardioactive peptide (CCAP) were used to elicit *I*_MI_ as shown before by Swensen and Marder (2000; see below). Proctolin was purchased from American Peptide or Bachem. CCAP was initially purchased from American Peptide, but in winter 2013 to spring 2014 this peptide did not produce *I*_MI_ values that were comparable to those reported in the literature (Swensen and Marder, 2000) and to our own previous results. Thus, all CCAP data from 2013-2014 were omitted, and CCAP used after that was obtained from Bachem. All other neuromodulators were obtained from Sigma-Aldrich.

### Solutions and drugs

W7, tetraethylammonium (TEA), dynasore, 1-(5-iodonaphthalene-1-sulfonyl)-1H-hexahydro-1,4-diazepine hydrochloride (ML-7), picrotoxin (PTX), Gallein, and fluphenazine came from Sigma-Aldrich. Bis(2-aminophenoxy)ethane-N,N,N',N'-tetra-acetic acid-acetoxymethyl ester (BAPTA-AM), tetrodotoxin (TTX), GTPγS, and sometimes W7, came from Tocris Bioscience. Calmidazolium, dantrolene, ryanodine, and pertussis toxin A protomer came from Enzo Life Sciences. CALP1 (sequence, VAITVLVK) and BSA came from Fisher Scientific. *N*-[2-[[[3-(4-chlorophenyl)-2-propenyl]methylamino]methyl]phenyl]-*N*-(2-hydroxyethyl)-4-methoxybenzenesulphonamide (KN-93) came from EMD Biosciences. All chemicals were aliquoted in either distilled H_2_O or DMSO and frozen until use.

### Measurement of *I*_MI_

Unless otherwise stated, all recordings of *I*_MI_ were made in the following standard recording saline solution: normal *Cancer* saline plus 0.1 μm TTX (to block sodium currents), 20 mm TEA (to block potassium currents), 10 μm PTX (to block synaptic currents), 5 mm CsCl (to block the H-current), and 200 μm CdCl_2_ (to block calcium currents). R568 experiments omitted CdCl_2_ as the combination of low calcium without BSA and CdCl_2_ depolarized cells, and reduced viability (data not shown). In some cells, spontaneous oscillations were observed under these conditions. When this happened, TTX and PTX concentrations were transiently raised to 1 and 30 μm, respectively, until oscillations stopped or were attenuated. Then, a normal solution was resumed for at least 20 min prior to the measurement of *I*_MI_.

Both proctolin and CCAP were bath applied at 1 μm for a volume of 5-10 ml at a perfusion rate of 3-4.5 ml/s. We also tried to use local pressure application of the peptides but found the responses to be too variable, and they were not used here for analysis (data not shown).

**Figure 1.**
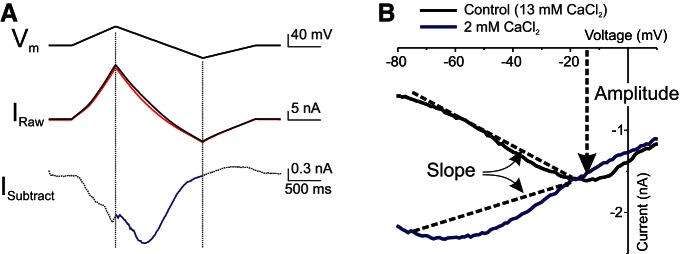
Modulator-activated *I*_MI_ measurement and quantification. ***A***, Proctolin (1 μm)-induced *I*_MI_ in LP neuron. Top, Voltage ramp protocol. Middle, Current in control ramp (black trace) and at the peak of the response to proctolin (red trace). Bottom, Difference current (blue trace) obtained by subtracting control currents from currents measured in proctolin. Only currents evoked during descending voltage ramps were considered (blue trace). ***B***, *I–V* curves of *I*_MI_ in normal saline solution (13 mm CaCl_2_, black trace) and low-calcium saline solution (2 mm CaCl_2_, blue trace). Changes in slope between −75 and −20 mV are used as a measure of *I*_MI_ voltage dependence. Amplitude at −15 mV is taken as a measure of *I*_MI_ activation. Note that *I*_MI_ in normal saline solution is close to maximal at this voltage, and there is minimal difference between normal calcium and low-calcium conditions.

All *I*_MI_ recordings were obtained in TEVC. A holding potential of −40 mV was used to prevent contamination from low-threshold activated potassium currents (Golowasch and Marder, 1992a). Figure 1*A* illustrates the procedure used to obtain *I*_MI_. The voltage was ramped from a holding potential of −40 mV, up to +20 mV, down to −80 mV, and back to −40 mV at 75 mV/s. The descending ramp was used to build *I–V* curves and to determine *I*_MI_ properties because it has been shown that the measurement of *I*_MI_ on descending ramps is less sensitive to ramp speed than ascending ramps (D. Fox, personal communication). These ramps were repeated every 35-45 s until no further changes in leak current were observed. At this point, the average of the last three to five ramps was defined as the control ramp (Fig. 1*A*, black trace). The average of three to five ramps at the peak of the response to neuromodulator was measured (Fig. 1*A*, red trace). The control currents were subtracted from the currents in the presence of neuromodulator, and this difference in current (Fig. 1*A*, blue trace) was defined as *I*_MI_. Any data that did not show a reversal of at least 50% of the *I*_MI_ maximum level upon washing were discarded.

### *I*_MI_ quantification

The following two features of *I*_MI_ were quantified from the *I–V* curves throughout: voltage dependence and activation level. Activation was quantified as the amplitude of the current at or near its peak (i.e., at −15 mV), and voltage dependence was quantified as the slope of the *I–V* curve between −75 and −20 mV (Fig. 1*B*). A value of −15 mV was chosen to estimate activation levels because, at this voltage, the amplitude of *I*_MI_ was not sensitive to changes in extracellular calcium [−1.2 ± 0.3, −2.7 ± 0.9, −2.1 ± 0.2, and −1.5 ± 0.5 nA, for calcium concentrations of 13, 6, 2, and 1 mm, respectively; one-way ANOVA for calcium concentrations on the second application (see rationale below): *F*_(3,20)_ = 2.37, *p* = 0.10].^e^ As described in the Results section, this enabled good separation of the effects of various agents on activation from those on voltage dependence. Additionally, this voltage is far enough from the estimated *I*_MI_ reversal potential (mean ± SD, +10.8 ± 9.8 mV, *n* = 43) to allow a reliable measurement of the current. In most of our experiments, *I*_MI_ did not reverse with the protocol used. The reversal potential was estimated only from currents that did reverse, and therefore this average reflects an underestimate of the *I*_MI_ reversal potential, even though it is more depolarized than previous reported estimates (i.e., −2.9 ± 14.7 mV; Golowasch and Marder, 1992b).

In order to evaluate the effects of pharmacological agents on signaling pathways potentially involved in *I*_MI_ activation by proctolin or CCAP, multiple applications of the peptides on each preparation were required. Therefore, we examined the effect of application number on *I*_MI_ amplitude and slope. Under normal calcium conditions, we observed that the first application was significantly larger at −15 mV^f^ and had a significantly more negative slope^g^ than the next four applications (Table 1). Amplitude or slope showed no significant differences from one another for applications two to five. Therefore, we used application two as our control application for all data reported here. However, in contrast to normal calcium conditions, when proctolin-induced *I*_MI_ was measured in 2 mm CaCl_2_ (a low-calcium solution), the amplitude remained unchanged during applications two and three, but dropped significantly with subsequent applications,^h^ while slope remained stable for applications two to five^i^ (Table 2). Therefore, low-calcium experiments were statistically analyzed with analysis of covariance (ANCOVA), a mixture of ANOVA and linear regression with drugs understudy as independent factors, and application number as the covariate. This method assumes that the desensitization rate can be predicted by application number, which we find to be true in low-calcium conditions, and is not affected by drug condition. As a consequence of this, *post hoc* testing could not be performed. This was not required for R568 data as only the first application in the low-calcium condition was used.

**Table 1:** Effect of proctolin application sequence on maximum amplitude and slope in normal calcium saline solution

Application 1		Application 2		Application 3		Application 4		Application 5	
Ampl (nA)	Slope (nS)	Ampl (nA)	Slope (nS)	Ampl (nA)	Slope (nS)	Ampl (nA)	Slope (nS)	Ampl (nA)	Slope (nS)
(*n* = 7)	(*n* = 7)	(*n* = 7)	(*n* = 7)	(*n* = 7)	(*n* = 7)	(*n* = 7)	(*n* = 7)	(*n* = 5)	(*n* = 5)
−2.8 ± 0.6*	−41 ± 10	−1.3 ± 0.3	−17 ± 4	−1.5 ± 0.3	−17 ± 5	−1.1 ± 0.3	−12 ± 3	−1.0 ± 0.3	−12 ± 6

All data were obtained from LP neurons. Ampl, Amplitude.

*Tukey’s test, *p* < 0.05 vs application 2.

**Table 2:** Effect of proctolin application sequence on maximum amplitude and slope in low-calcium saline solution

Application 2		Application 3		Application 4		Application 5		Application 6	
Ampl (nA)	Slope (nS)	Ampl (nA)	Slope (nS)	Ampl (nA)	Slope (nS)	Ampl (nA)	Slope (nS)	Ampl (nA)	Slope (nS)
(*n* = 3)	(*n* = 3)	(*n* = 3)	(*n* = 3)	(*n* = 3)	(*n* = 3)	(*n* = 3)	(*n* = 3)	(*n* = 3)	(*n* = 3)
−2.1 ± 0.9	+26 ± 10	−1.6 ± 0.7	+23 ± 11	−1.1 ± 0.6*	+19 ± 5	−0.8 ± 0.6**	+22 ± 6	−0.7 ± 0.5**	+18±6

Data are reported as the mean ± SEM. First application was not included as it was performed in normal calcium conditions. All data obtained from LP neurons. Ampl, Amplitude.

*Tukey’s test, *p* < 0.05 vs application 2 of same calcium condition. **Tukey test’s, *p* < 0.01 vs application 2 of same calcium condition.

### Statistics and data analysis

All calculations of *I*_MI_, and measurements of difference currents and leak subtractions were performed with the pClamp version 9 or 10.4 (Molecular Devices) family of software. All data were digitally filtered after acquisition using an eight-pole Bessel filter with a cutoff frequency of 320 Hz. Data were reduced so that the currents measured during a voltage ramp were divided into 1 mV bins (of ∼13.3 ms each). These data were stored in Microsoft Excel files for databasing and graph making. All statistics were performed with SigmaPlot 11, except for those analyses that required ANCOVA, which were performed with IBM SPSS Statistics 22. Some data were found to be not normal and/or not homoscedastic. For this reason, for two-group comparisons, Mann–Whitney rank sum tests were run. In multiple-group comparisons that failed normality/equal variance testing, the dependent variable was ranked, and statistics were run on this rank score. All data after this transformation passed either an equal variance test (*F* test, SigmaPlot) or Levene’s test (SPSS). If data passed normality testing (Shapiro–Wilk test, *p* > 0.05) after a transformation, statistical testing was performed on the ranked dependent variable. If data failed normality testing after transformation, all statistical testing was performed on the original data. No data failed equal variance testing after rank transformation. All graphs making comparisons between conditions show the average ± SEM, unless noted otherwise. For the majority of experiments, controls and drug applications were performed on alternating days to reduce any biases. Table 4 summarizes the statistical tests (normality, type of test, and *post hoc* power) and the conditions used in all of the data reported.

## Results

The reduction of the voltage dependence of *I*_MI_ in low-calcium conditions (Fig. 1*B*) was originally interpreted by Golowasch and Marder (1992b) as the removal of a voltage-dependent extracellular block of the modulator-activated channels by calcium (similar to the voltage-dependent effect of magnesium on NMDA channels; Nowak et al., 1984). Swensen and Marder (2000) observed that the calmodulin blocker W7 enhanced *I*_MI_ in a voltage-dependent manner and concluded that a calmodulin-dependent pathway was likely involved in *I*_MI_ activation. An alternate interpretation of those results is that the current was not enhanced but instead lost voltage dependence, in a manner similar to the loss of voltage dependence in a low-calcium saline solution, thus explaining why the current was enhanced more at −80 mV than it was at −40 mV (Swensen and Marder, 2000). As calmodulin is a ubiquitous calcium sensor, we hypothesized that calmodulin may mediate *I*_MI_ voltage dependence. According to this model, in normal calcium conditions, high levels of calcium influx through calcium channels, or perhaps through *I*_MI_ itself, would keep calmodulin in a relatively activated state. This activated calmodulin would either bind directly to the channel itself or modify *I*_MI_ channels indirectly via calmodulin-activated proteins from a voltage-independent to a voltage-dependent state. When extracellular calcium is reduced, there is less activated calmodulin and therefore reduced voltage dependence. We therefore predicted that full *I–V* relations in the presence of calmodulin inhibitors should reveal a reduced voltage dependence.

### The calmodulin inhibitor W7 reduces proctolin and CCAP-induced *I*_MI_ voltage dependence

**Figure 2.**
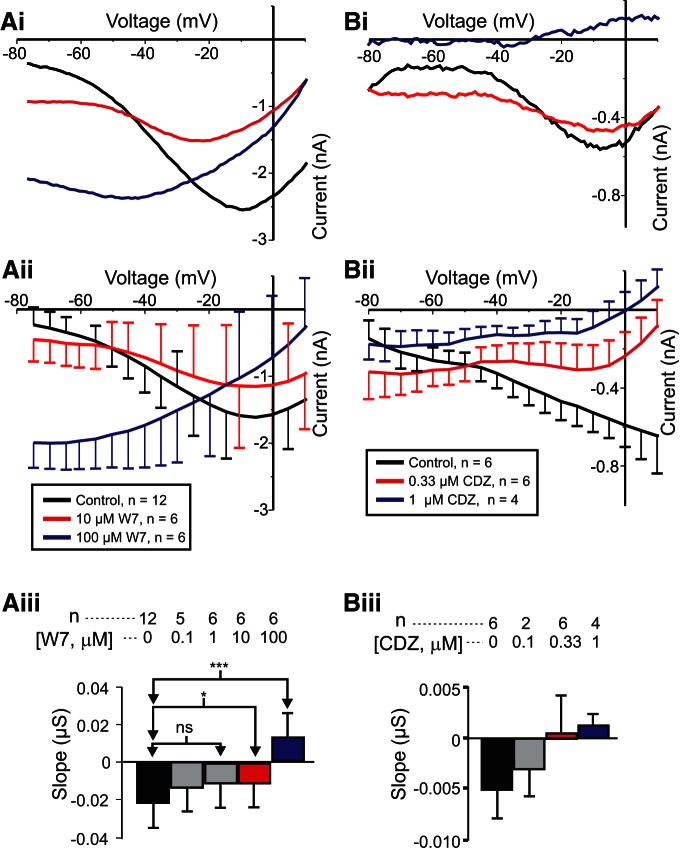
The effect of calmodulin inhibitors on *I*_MI_ voltage dependence. Left, Proctolin-induced *I*_MI_ at different concentrations of W7. ***Ai***, Representative *I–V* curves of a W7 experiment. ***Aii***, Averaged *I–V* curves across W7 experiments. ***Aiii***, Quantification of W7 data. A one-way repeated-measures ANOVA showed that W7 changed the proctolin-induced *I*_MI_ slope (*F*_(4,19)_ = 15.972, *p* = 6.96 × 10^−6j^). Error bars indicate the SEM. Tukey’s test; *p < 0.05; ***p < 0.001. Right, Proctolin-induced *I*_MI_ in different concentrations of calmidazolium (CDZ). ***Bi***, Representative *I–V* curves. ***Bii***, Average *I–V* curves from all calmidazolium experiments. ***Biii***, Quantification of all calmidazolium data. A one-way repeated-measures ANOVA showed that calmidazolium significantly altered *I*_MI_ slope (*F*_(3,9)_ = 4.846, *p* = 0.028^n^). However, no significant *post hoc* pairwise differences were observed. Tukey’s *post hoc* test, *p* < 0.05. Error bars indicate the SEM. Data are from LP neurons.

To test the hypothesis that activated calmodulin mediates *I*_MI_ voltage dependence, we measured *I*_MI_ in the presence and absence of the calmodulin inhibitor W7, predicting that W7 should increase *I*_MI_ slope in a dose-dependent manner. As shown in Figure 2, *Ai* and *Aii*, proctolin-induced *I*_MI_ exhibited a negative slope when measured before W7 exposure (black trace). However, at both 10 µm W7 (Fig. 2*Ai*,*Aii*, red trace) and 100 µm W7 (Fig. 2*Ai*,*Aii*, blue trace), this slope becomes increasingly more positive. When these experiments were repeated at various concentrations of W7, it was found that W7 increased proctolin-induced *I*_MI_ slope in a dose-dependent manner. A one-way repeated-measures ANOVA showed that W7 significantly changed the *I*_MI_ slope (*F*_(4,19)_ = 15.972, *p* = 6.96 × 10*^−^* ^6j^). A *post hoc* Tukey’s test showed that these changes were significant at concentrations of ≥10 µm (Fig. 2*Aiii*). Interestingly, a one-way repeated-measures ANOVA showed that W7 changed proctolin-induced *I*_MI_ amplitude at −15 mV (*F*_(4,19)_ = 3.243, *p* = 0.035^k^). However, this appeared to be a biphasic effect, because *post hoc* tests revealed that only at a concentration of 10 µm was the amplitude significantly different from that of controls, but not at higher concentrations (data not shown). We also replicated these results for 1 µm CCAP (control slope = −0.013 ± 0.003 µS; 33 µm W7 slope = −0.003 ± 0.003; paired Student’s *t* test (*t*_(5)_ = −2.625, *p* = 0.047, *n* = 6^l^; see also Fig. 8). In contrast, no significant effect on amplitude at −15 mV was found for CCAP-induced *I*_MI_ (paired Student’s *t* test: *t*_(5)_ = −0.435, *p* = 0.681, *n* = 6^m^), but concentrations of W7 <33 µm were not tested for CCAP-induced *I*_MI_. The findings for CCAP-activated *I*_MI_ support the hypothesis that activated calmodulin mediates *I*_MI_ voltage dependence.

### The calmodulin inhibitor calmidazolium reduces *I*_MI_ voltage dependence

Although W7 increases neuromodulator-induced *I*_MI_ slope, and it has been shown to inhibit calmodulin-dependent effects in other crab species (Peracchia, 1987), it is known to also have nonspecific effects on calmodulin-activated proteins and calmodulin-like proteins. For example, W7 has been shown to directly inhibit calmodulin-activated proteins themselves, such as myosin light chain kinase (MLCK), independent of calmodulin activity (Inagaki et al., 1986; Saitoh et al., 1987), and it has been used both to purify (Todoroki et al., 1991) and to inhibit (Millward et al., 1998) other EF-hand domain proteins. Thus, having no known direct assay for calmodulin function in this system, we decided to use structurally different calmodulin inhibitors, and selected non-naphthalene–sulfonamide calmodulin inhibitors fluphenazine and calmidazolium. The D_1_/D_2_ receptor antagonist, and calmodulin inhibitor, fluphenazine appeared to be toxic to our cells at concentrations far below those required for calmodulin inhibition (1 mm) in crayfish muscle (Sedlmeier and Dieberg, 1983; *n* = 3; data not shown). Calmidazolium showed no toxic effects and increased *I*_MI_ slope in a dose-dependent manner (Fig. 2*Bi*,*Bii*). A one-way repeated-measures ANOVA showed that calmidazolium significantly altered *I*_MI_ slope (*F*_(3,9)_ = 4.846, *p* = 0.028^n^), although *post hoc* analysis did not show specific concentration effects (Fig. 2*Biii*). This is consistent with the hypothesis that activated calmodulin mediates *I*_MI_ voltage dependence. Interestingly, a one-way repeated-measures ANOVA test showed that calmidazolium also significantly altered *I*_MI_ amplitude at −15 mV (*F*_(3,9)_ = 6.382, *p* = 0.013^o^). This reduction in *I*_MI_ amplitude is consistent with that observed for proctolin-induced *I*_MI_ at 10 µm concentration of W7 shown previously. All three experiments (W7 on both proctolin and CCAP-activated *I*_MI_, and calmidazolium on proctolin-activated *I*_MI_), however, showed a consistent inhibitory effect on slope, supporting the hypothesis that activated calmodulin mediates both proctolin- and CCAP-induced *I*_MI_ voltage dependence.

### The calmodulin agonist CALP1 does not restore *I*_MI_ voltage dependence in low calcium

As the preceding results suggested that activated calmodulin mediated the voltage dependence of *I*_MI_, we predicted that calmodulin agonists should be able to restore *I*_MI_ voltage dependence in a low-extracellular calcium solution. We applied the calmodulin activator CALP1, a membrane-permeable peptide that locks the EF-hand domains of calmodulin in the “on state,” thus bypassing the requirement for calcium (Manion et al., 2000), with the prediction that this should make the *I*_MI_ slope more negative in low-calcium solutions. Two hour incubations of CALP1 at 1 µm (*n* = 4), 10 µm (*n* = 4), and 50 µm (*n* = 2) showed no significant effect on proctolin-induced *I*_MI_ slope (one-way ANOVA: *F*_(3,16)_ = 1.077, *p* = 0.387)^p^ or amplitude (one-way ANOVA: *F*_(3,16)_ = 0.437, *p* = 0.729)^q^ in a low-calcium saline solution (Table 3). Similarly, when this experiment was repeated with overnight incubations in 50 µm CALP1 (*n* = 3), no difference was observed compared with control solutions (*n* = 2) for either slope (Student’s *t* test: *t*_(3)_ = 0.44, *p* = 0.689)^r^ or amplitude (Student’s *t* test: *t*_(3)_ = 0.777, *p* = 0.494^s^). We do not have a satisfactory explanation for these results at this point, except that CALP1 may not be effective in crustaceans or that, once calmodulin is dislodged from the receptor because of the low-calcium condition, it cannot be reactivated by CALP1 (see Discussion).

**Table 3:** Drugs that affected *I*_MI_ slope

Drug name	[Drug]	[Ca]	Neuromodulator	*n*	*I*_MI_ slope (nS)
W7	0 µm	13 mm	Proctolin	12	−21.7 ± 3.7
	0.1 µm	13 mm	Proctolin	5	−13.5 ± 4.2
	1 µm	13 mm	Proctolin	6	−11.2 ± 2.4*
	10 µm	13 mm	Proctolin	6	−11.4 ± 3.9**
	100 µm	13 mm	Proctolin	6	+13.1 ± 3.4***
Calmidazolium	0 µm	13 mm	Proctolin	6	−20.4 ± 10.6
	0.1 µm	13 mm	Proctolin	2	−13.7 ± 11.1
	0.3 µm	13 mm	Proctolin	6	+1.6 ± 14
	0.0 µm	13 mm	Proctolin	4	+4.3 ± 4.9
Dantrolene	0 µm	13 mm	Proctolin	6	−8.1 ± 3.2
	3.3 µm	13 mm	Proctolin	6	+1.6 ± 1.9**
CALP1	0 µm	2 mm	Proctolin	11	+18.2 ± 4.9
	1 µm	2 mm	Proctolin	4	+10.2 ± 6.4
	10 µm	2 mm	Proctolin	4	+28.2 ± 3.8
	50 µm	2 mm	Proctolin	2	+23.5 ± 9.4
KN-93	0 µm	13 mm	Proctolin	7	−21 ± 4.2
	Low dose	13 mm	Proctolin	5	+0.8 ± 5.8
	High dose	13 mm	Proctolin	2	+16.6 ± 2.2
Pertussis toxin	0 µg/ml	13 mm	Proctolin	7	−3.1 ± 2.9
	0 µg/ml	2 mm	Proctolin	6	+14.4 ± 3.1
	10 µg/ml	13 mm	Proctolin	7	+1.5 ± 2.9
	10 µg/ml	2 mm	Proctolin	6	+21.5 ± 3.1
GTPγS	0 mm	13 mm	Proctolin	9	−2.2 ± 2.9
	0 mm	2 mm	Proctolin	8	+11.5 ± 3.1
	10 mm	13 mm	Proctolin	5	−6.7 ± 4.0
	10 mm	2 mm	Proctolin	4	+15.2 ± 4.4
Gallein	0 µm	13 mm	Proctolin	9	−5.4 ± 1.6
	1 µm	13 mm	Proctolin	9	+1.5 ± 1.9*
	3 µm	13 mm	Proctolin	9	+0.7 ± 2.8
ML-7	0 µm	13 mm	Proctolin	12	−4.2 ± 1.5
	0.1 µm	13 mm	Proctolin	12	+1.7 ± 2.3*
	1 µm	13 mm	Proctolin	10	+4.5 ± 1.4***
	10 µm	13 mm	Proctolin	7	+3.1 ± 2.4*
Dynasore, W7	0, 0 µm	13 mm	CCAP	18	−11.3 ± 1.3
	0, 33 µm	13 mm	CCAP	6	−0.4 ± 2.3***
	33, 0 µm	13 mm	CCAP	6	−12.6 ± 2.3
	33, 33 µm	13 mm	CCAP	6	−7.7 ± 2.3†
R568	0 µm	13 mm	Proctolin	10	−0.7 ± 4.5
	0 µm	2 mm	Proctolin	6	+16.5 ± 5.9
	10 µm	13 mm	Proctolin	4	+17.3 ± 7.2*
	10 µm	2 mm	Proctolin	4	+36.9 ± 7.2‡

Data are reported as the mean ± SEM. All data were obtained from LP neurons.

Tukey’s test/*t* test *p* value vs control: *p < 0.05, **p < 0.01, ***p < 0.001; †p < 0.05 vs W7 alone; ‡*p* < 0.05 vs low-calcium control.

**Table 4:** Statistical tests

Letter, experiment name	Data structure (distribution)	Type of test	Power (α = 0.05)
^a^ Low-calcium effect on RMP	Normal	Two-tailed paired *t* test	1.00
^b^ Low-calcium effect on R_in_	Normal	Two-tailed paired *t* test	0.84
^c^ BSA effect on low calcium-induced depolarization	Normal	Two-tailed *t* test	1.00
^d^ BSA effect on low calcium-induced effects of R_in_	Non-normal	Mann–Whitney rank sum test	0.17*
^e^ Effect of low calcium on I_MI_ amplitude	Normal	One-way ANOVA	0.30
^f^ Effect of application number on I_MI_ amplitude in normal calcium	Normal	One-way ANOVA, post hoc Tukey’s tests	0.81
^g^ Effect of application number on slope in normal calcium	Normal	One-way ANOVA, post hoc Tukey’s tests	0.81
^h^ Effect of application number on I_MI_ amplitude in low calcium	Normal	Two-way repeated-measures ANOVA, post hoc Tukey’s tests	Calcium = 0.05Application = 0.90Interaction = 0.59
^i^ Effect of application number on slope in low calcium	Normal	Two-way repeated-measures ANOVA, post hoc Tukey’s tests	Calcium = 0.94Application = 0.05Interaction = 0.13
^j^ W7 effect on proctolin-induced I_MI_ slope	Normal	One-way repeated-measures ANOVA, post hoc Tukey’s tests	1.00
^k^ W7 effect on proctolin-induced I_MI_ amplitude	Normal	One-way repeated-measures ANOVA, post hoc Tukey’s tests	0.54
^l^ W7 effect on CCAP-induced I_MI_ slope	Normal	Two-tailed paired t test	0.50
^m^ W7 effect on CCAP-induced I_MI_ amplitude	Normal	Two-tailed paired t test	0.05
^n^ Effect of calmidazolium on proctolin-induced I_MI_ slope	Normal	One-way repeated-measures ANOVA, post hoc Tukey’s tests	0.62
^o^ Effect of calmidazolium effect on proctolin-induced I_MI_ amplitude	Normal	One-way repeated-measures ANOVA, post hoc Tukey’s tests	0.78
^p^ Effect of CALP1 (2 h) on proctolin-induced I_MI_ slope	Normal	One-way ANOVA	0.07
^q^ Effect of CALP1 (2 h) on proctolin-induced I_MI_ amplitude	Normal	One-way ANOVA	0.05
^r^ Effect of CALP1 (overnight) on proctolin-induced I_MI_ slope	Normal	Two-tailed t test	0.10
^s^ Effect of CALP1 (overnight) on proctolin-induced I_MI_ amplitude	Normal	Two-tailed t test	0.10
^t^ Dantrolene effect on proctolin-induced I_MI_ slope	Normal	Two-tailed paired t test	0.91
^u^ Dantrolene effect on proctolin-induced I_MI_ amplitude	Normal	Two-tailed paired t test	0.77
^w^ KN-93 effect on proctolin-induced I_MI_ slope	Normal	One-way repeated-measures ANOVA, post hoc Tukey’s tests	1.00
^x^ KN-93 effect on proctolin-induced I_MI_ amplitude	Normal	One-way repeated-measures ANOVA, post hoc Tukey’s tests	1.00
^y^ Effect of gallein on proctolin-induced I_MI_ slope	Normal	One-way repeated-measures ANOVA, post hoc Tukey’s tests	0.56
^z^ Effect of gallein on proctolin-induced I_MI_ amplitude	Normal	One-way repeated-measures ANOVA, post hoc Tukey’s tests	0.13
^aa^ Effect of NPS-2143 on proctolin-induced I_MI_ slope	Normal	One-way repeated-measures ANOVA, post hoc Tukey’s tests	0.57
^ab^ Effect of NPS-2143 on proctolin-induced I_MI_ amplitude	Normal	One-way repeated-measures ANOVA, post hoc Tukey’s tests	0.06
^ac^ ML7 effect on proctolin-induced I_MI_ slope	Normal	One-way repeated-measures ANOVA, post hoc Tukey’s tests	0.95
^ad^ ML7 effect on proctolin-induced I_MI_ amplitude	Normal	One-way repeated-measures ANOVA, post hoc Tukey’s tests	0.71
^ae^ Effect of dynasore on W7’s reduction of I_MI_ voltage dependence	Normal	Two-way ANOVA, post hoc Tukey’s tests	W7 = 0.99Dynasore = 0.55Interaction = 0.26
^af^ Effect of dynasore on effect of W7 on I_MI_ amplitude	Normal	Two-way ANOVA, post hoc Tukey’s tests	W7 = 0.88Dynasore = 0.10Interaction = 0.17
^ag^ R568 effect on proctolin-induced I_MI_ slope	Normal	Two-way ANOVA, post hoc Tukey’s tests	Calcium = 0.86R568 = 0.89Interaction = 0.10
^ah^ R568 effect on proctolin-induced I_MI_ amplitude	Non-normal	Two-way ANOVA, post hoc Tukey’s tests	Calcium = 1.00R568 = 1.00Interaction = 0.70

All data obtained from LP neurons. RMP is resting membrane potential, Rin is input resistance.

*Post hoc power calculation performed for *t* test.

### Intracellular calcium effects

Our hypothesis that calcium-activated calmodulin mediates *I*_MI_ voltage dependence can be tested (indirectly) by lowering the intracellular calcium concentration, with the prediction that this will reduce activated calmodulin level and in turn decrease *I*_MI_ voltage dependence (i.e., increase the *I*_MI_ slope).

#### The calcium chelator BAPTA-AM does not alter *I*_MI_ or the transient high-threshold potassium current

In order to lower intracellular calcium concentrations, we tried both incubating preparations in 30 µm membrane-permeable calcium chelator BAPTA-AM for 2 h (*n* = 6), and overnight (∼18 h) at 30 µm (*n* = 3) and 100 µm (*n* = 5). In all of these experiments, no significant changes in *I*_MI_ slope or amplitude were observed (data not shown). Surprisingly, we also saw no change in the high-threshold potassium current (*I*_HTK_). *I*_HTK_ is dominated by a large Ca-dependent potassium current (Graubard and Hartline, 1991; Golowasch and Marder, 1992a) and was thus expected to be greatly affected. We conclude that BAPTA-AM may be ineffectual in these cells (cells may lack the intracellular esterases or BAPTA may be locked in the wrong intracellular compartment after de-esterification) or its buffering capacity may be too small to handle the large influx of calcium in this system. These observations are consistent with similar observations in *C. borealis* cardiac ganglion neurons in which *I*_HTK_ was also unaffected by BAPTA-AM (Ransdell et al., 2012). Our attempts to inject BAPTA directly into a different cell type, the pyloric dilator, PD, cell by iontophoresis (*n* = 3) produced inconsistent and inconclusive results, presumably because of the effects of current injection on *I*_HTK_, such as those reported by Golowasch et al. (1999).

#### The ryanodine receptor antagonist dantrolene reduces *I*_MI_ voltage dependence

**Figure 3.**
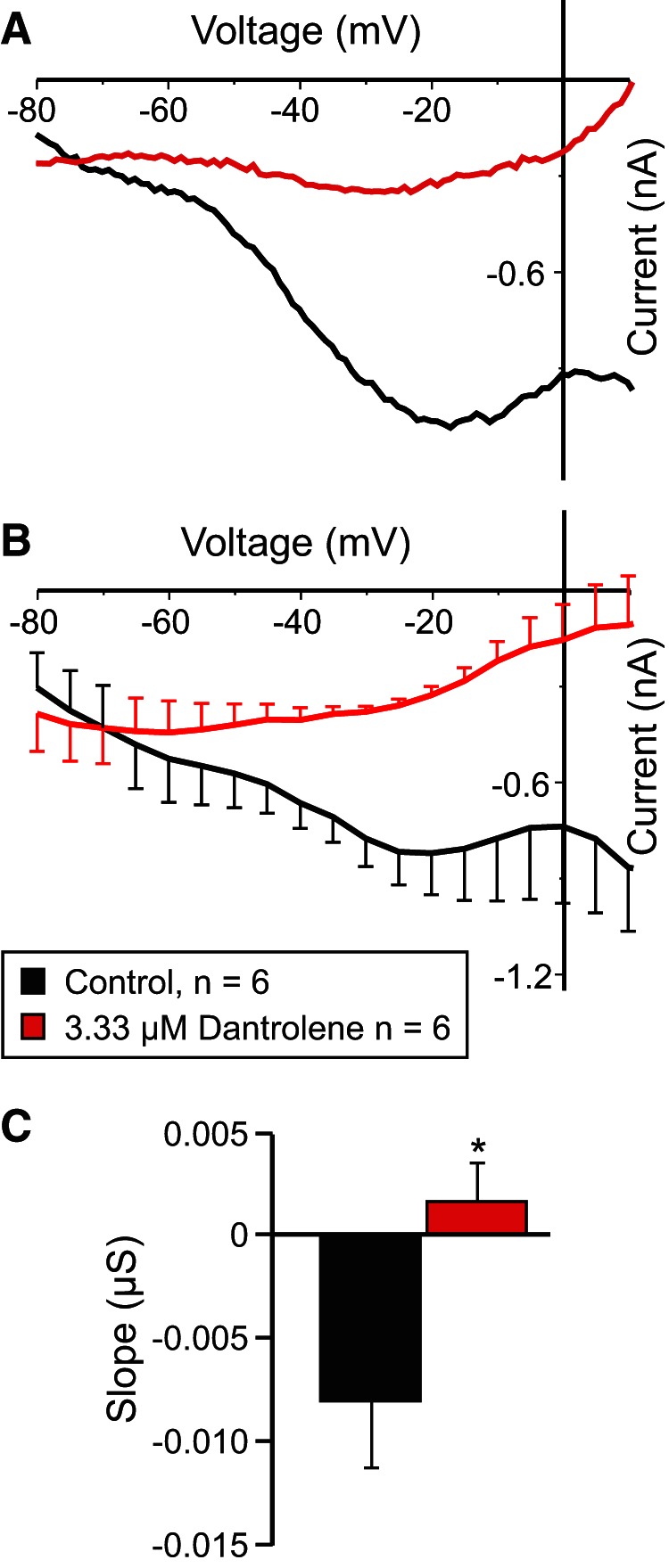
The ryanodine antagonist dantrolene reduces *I*_MI_ voltage dependence. ***A***, Representative *I–V* curve of the proctolin-induced *I*_MI_ before (black trace) and after (red trace) application of 3.33 μm dantrolene. ***B***, Averaged *I–V* curves of all dantrolene experiments. ***C***, Quantification of dantrolene data shown in ***B***. A paired two-tailed Student’s *t* test showed that dantrolene significantly increased proctolin-induced *I*_MI_ slope (*t*_(5)_ = −4.230, *p* = 0.008^t^). *p < 0.05. Error bars indicate the SEM. Recordings are from LP neurons.

We found that incubation with the ryanodine receptor antagonist dantrolene, a known inhibitor of ryanodine receptors in crustaceans (Olivares et al., 1993; Porras et al., 2001) was effective in changing the *I*_MI_ slope. As ryanodine receptors mediate calcium-induced calcium release (Zhao et al., 2001), incubation in dantrolene was expected to lower intracellular calcium levels and thus reduce *I*_MI_ voltage dependence. Indeed, incubation in 3.33 µm dantrolene reduced proctolin-induced *I*_MI_ voltage dependence (Fig. 3). Figure 3*A* shows a representative example, and Figure 3*B* shows the averages from a set of six experiments. A paired two-tailed Student’s *t* test showed that dantrolene significantly increased *I*_MI_ slope (*t*_(5)_ = −4.230, *p* = 0.008^t^; Fig. 3*C*). This is consistent with the hypothesis that calcium-activated calmodulin is necessary for *I*_MI_ voltage dependence. Dantrolene also significantly decreased *I*_MI_ amplitude at −15 mV (paired Student’s *t* test: *t*_(5)_ = −3.502, *p* = 0.017^u^). These results are consistent with our previous calmodulin inhibitor results, which suggest a dual role for intracellular calcium and calmodulin in the regulation of *I*_MI_ voltage dependence and activation.

### Role of calmodulin-activated kinases in *I*_MI_ voltage dependence

**Figure 4.**
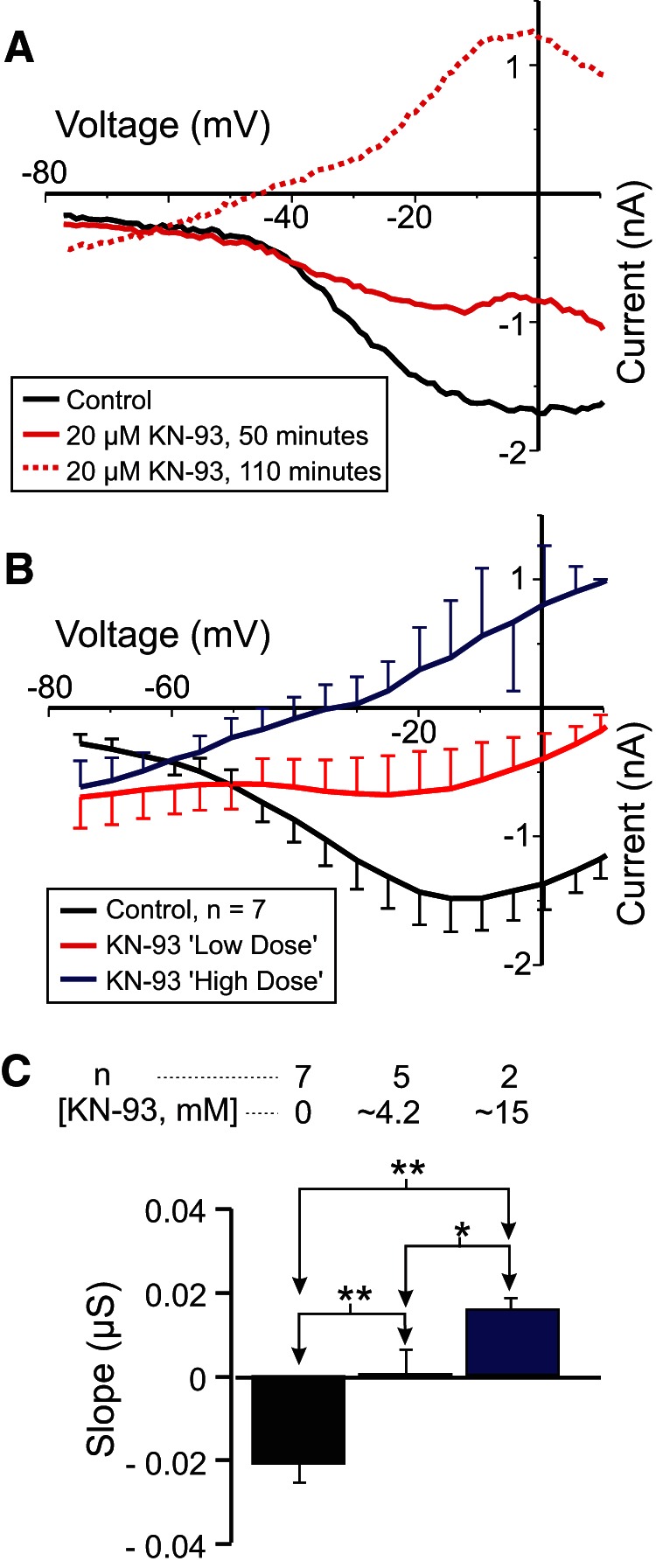
The CaMKII inhibitor KN-93 reduces *I*_MI_ voltage dependence. Proctolin-induced *I*_MI_ at different concentrations of KN-93. For statistical analysis, KN-93 was grouped into a low-dose (2-5 μm) and a high-dose (10-20 μm) group, in addition to a control group (0 μm). ***A***, Representative *I–V* curves for a KN-93 experiment. ***B***, Averaged *I–V* curves for all KN-93 experiments. ***C***, Quantification of data shown in ***B***. A one-way repeated-measures ANOVA showed that KN-93 significantly increased proctolin-induced *I*_MI_ slope (*F*_(2,5)_ = 46.239, *p* = 5.96 × 10^−4w^). Recordings are from LP neurons.

In order to determine whether calmodulin-activated proteins play a role in *I*_MI_ voltage dependence, we investigated inhibitors of calmodulin-activated kinases and phosphatases. In fact, the best known case of voltage dependence mediated by an intracellular signaling cascade is that of “on” bipolar cells of dogfish retina that was described by Shiells and Falk (2000) and appears to require the activation of CaMKII. Therefore, we first examined the effects of the membrane-permeable CaMKII inhibitor KN-93, an inhibitor that has been successfully used in crayfish neurons (Uzdensky et al., 2007). We predicted that KN-93 should reduce *I*_MI_ voltage dependence. As shown in Figure 4, *A* and *B*, KN-93 applied for ∼2 h reduced proctolin-induced *I*_MI_ voltage dependence in a dose-dependent manner. Figure 4*A* illustrates the gradual increase in slope observed 50 min after washout (solid green trace) and then 110 min after washout (dashed green trace) from KN-93 application. For statistical analysis, due to the low sample number, these groups were divided into a control condition, a “low-dose” KN-93 condition consisting of the combined data for KN-93 at 2 μm (*n* = 1), 4 μm (*n* = 1), and 5 μm (*n* = 3), and a “high dose” KN-93 condition corresponding to the combined data for KN-93 at 10 μm (*n* = 1) and 20 μm (*n* =1). A one-way repeated-measures ANOVA showed that KN-93 significantly increased proctolin-induced *I*_MI_ slope (*F*_(2,5)_ = 46.24, *p* = 5.96 × 10^−4w^). A *post hoc* Tukey’s test shows that both the low-dose (*p* = 0.004) and high-dose (*p* = 0.002) conditions were significantly different from control and from one another (*p* = 0.043). On the other hand, a one-way repeated-measures ANOVA also showed that KN-93 significantly decreased the amplitude of proctolin-induced *I*_MI_ at −15 mV (*F*_(2,5)_ = 34.09, *p* = 0.001),^x^ with a *post hoc* Tukey’s test showing that both the low dose (*p* = 0.013) and high dose (*p* = 0.003) conditions were significantly different from control and from one another (*p* = 0.034). Combined with the finding that a CaMKII-like enzyme has been identified in the stomatogastric nervous system of crustaceans (Withers et al., 1998), our results suggest that CaMKII plays a dual role in both *I*_MI_ voltage dependence and activation.

### The calcium-sensing receptor hypothesis

The results reported so far are clearly not consistent with the original hypothesis of Golowasch and Marder (1992b) of an extracellular voltage-dependent block of *I*_MI_ channels by calcium. Instead, it indicates a clear role of extracellular calcium mediated by a calmodulin-dependent mechanism.

We hypothesized that the activation by extracellular calcium of CaSRs, which are known to require binding of activated calmodulin for their stable expression on the cell surface (Huang et al., 2010), might explain the voltage dependence of *I*_MI_. CaSRs are known to be G-protein-coupled receptors of the metabotropic glutamate receptor family (Conigrave et al., 2007; Conigrave and Hampson, 2010; Huang et al., 2010), and we propose that the activation of such a G-protein-coupled CaSR provides a voltage dependence signal to *I*_MI_ (see Fig. 10). According to this model, in a low-calcium saline solution, the CaSR ligand is missing, which leads to the observed loss of voltage dependence. This would also explain the loss of voltage dependence in the presence of calmodulin inhibitors, which are known to destabilize the receptor and lead to its subsequent endocytosis (Huang et al., 2010). Below, we test this hypothesis.

### The βγ-subunit inhibitor gallein decreases *I*_MI_ voltage dependence

**Figure 5.**
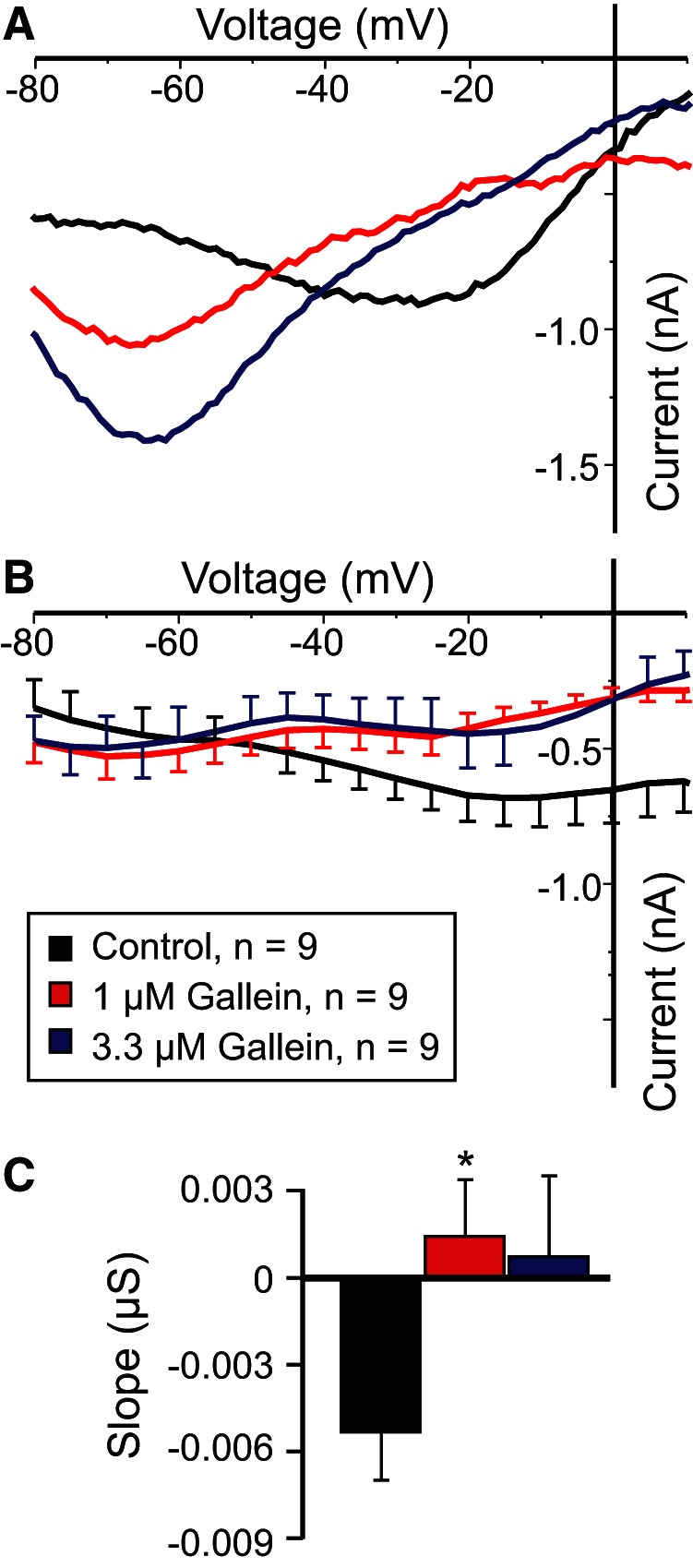
The Gβγ-subunit inhibitor gallein increases *I*_MI_ slope. ***A***, Representative *I–V* curves of proctolin-induced *I*_MI_ in gallein. ***B***, Averaged *I–V* curves of proctolin-induced *I*_MI_ for all gallein experiments. ***C***, Quantification of the data shown in ***A***. ***B***, A one-way repeated-measures ANOVA showed that gallein significantly increased proctolin-induced *I*_MI_ slope (*F*_(2,16)_ = 4.445, *p* = 0.029^y^). Error bars indicate the SEM. Tukey’s test, *p < 0.05. Recordings are from LP neurons.

The CaSR is a member of the (class 3/C) G-protein-coupled metabotropic glutamate receptor family, typically associated with G_q_ or G_i_ signaling (Conigrave et al., 2007; Conigrave and Hampson, 2010; Huang et al., 2010). No commercially available inhibitors of G_q_ are currently available. Thus, we targeted G_i_ by using the G_o_/G_i_ inhibitor pertussis toxin as well as the nonhydrolyzable GTP derivative GTPγS and detected no changes in the slope of *I*_MI_ (Table 3). Similarly, we saw no evidence that G_i_ was involved, because the pertussis toxin did not produce changes to the *I*_MI_ slope (Table 3). To test the possibility that the Gβγ-subunits may be involved, we examined the effects of the inhibitor gallein and found that it significantly reduced the proctolin-induced *I*_MI_ voltage dependence (Fig. 5*A*,*B*). As shown in Figure 5, *A* and *B*, increasing concentrations of gallein increased proctolin-induced *I*_MI_ slope in a dose-dependent manner (Table 3). A one-way repeated-measures ANOVA showed that this effect was statistically significant (*F*_(2,16)_ = 4.445, *p* = 0.029^y^), with concentrations of 1 μm already at saturation level (Fig. 5*C*). Importantly, this modulation appeared to be independent of *I*_MI_ activation, because a one-way repeated-measures ANOVA showed that gallein did not significantly change *I*_MI_ amplitude at −15 mV (*F*_(2,16)_ = 2.337, *p* = 0.129^z^). These results suggest that *I*_MI_ voltage dependence may be modulated by G-protein-coupled receptors, specifically via βγ-subunits of heterotrimeric G-proteins.

### The specific CaSR antagonist NPS-2143 reduces *I*_MI_ voltage dependence

**Figure 6.**
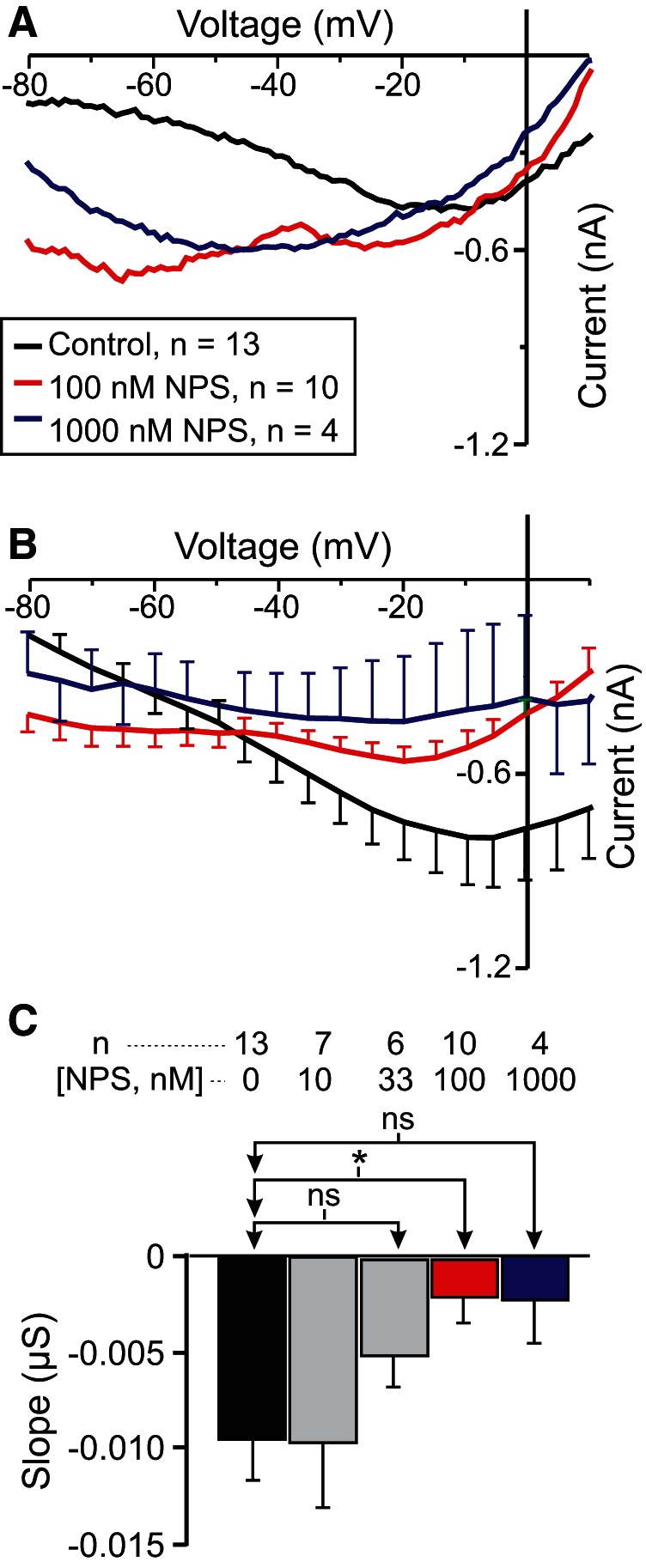
The specific CaSR antagonist NPS-2143 increases *I*_MI_ slope in a normal calcium condition in LP neurons. ***A***, Representative *I–V* curves showing the effect of NPS-2143 (NPS) at different concentrations on proctolin-induced *I*_MI_. ***B***, Averaged *I–V* curves of all NPS-2143 experiments. ***C***, Quantification of all NPS-2143 data. A one-way repeated-measures ANOVA showed that NPS-2143 significantly altered proctolin-induced *I*_MI_ slope (*F*_(4,22)_ = 3.314, *p* = 0.029^aa^). Error bars indicate the SEM. Tukey’s test, *p < 0.05.

If *I*_MI_ voltage dependence is due to signaling by CaSR, then the specific CaSR antagonist NPS-2143 should increase *I*_MI_ slope in a dose-dependent manner. As shown in Figure 6, *A* and *B*, NPS-2143 applied in the presence of normal calcium levels increased proctolin-induced *I*_MI_ slope. A one-way repeated-measures ANOVA showed that NPS-2143 significantly altered proctolin-induced *I*_MI_ slope (*F*_(4,22)_ = 3.314, *p* = 0.029^aa^; Fig. 6*C*, Table 3). In contrast, a one-way repeated-measures ANOVA showed that NPS-2143 did not significantly alter *I*_MI_ amplitude at −15 mV [*F*_(4,22)_ = 1.085, *p* = 0.388^ab^). These results are consistent with the hypothesis that *I*_MI_ voltage dependence is mediated by active detection of extracellular calcium by a CaSR.

### The MLCK inhibitor ML-7 reduces *I*_MI_ voltage dependence

**Figure 7.**
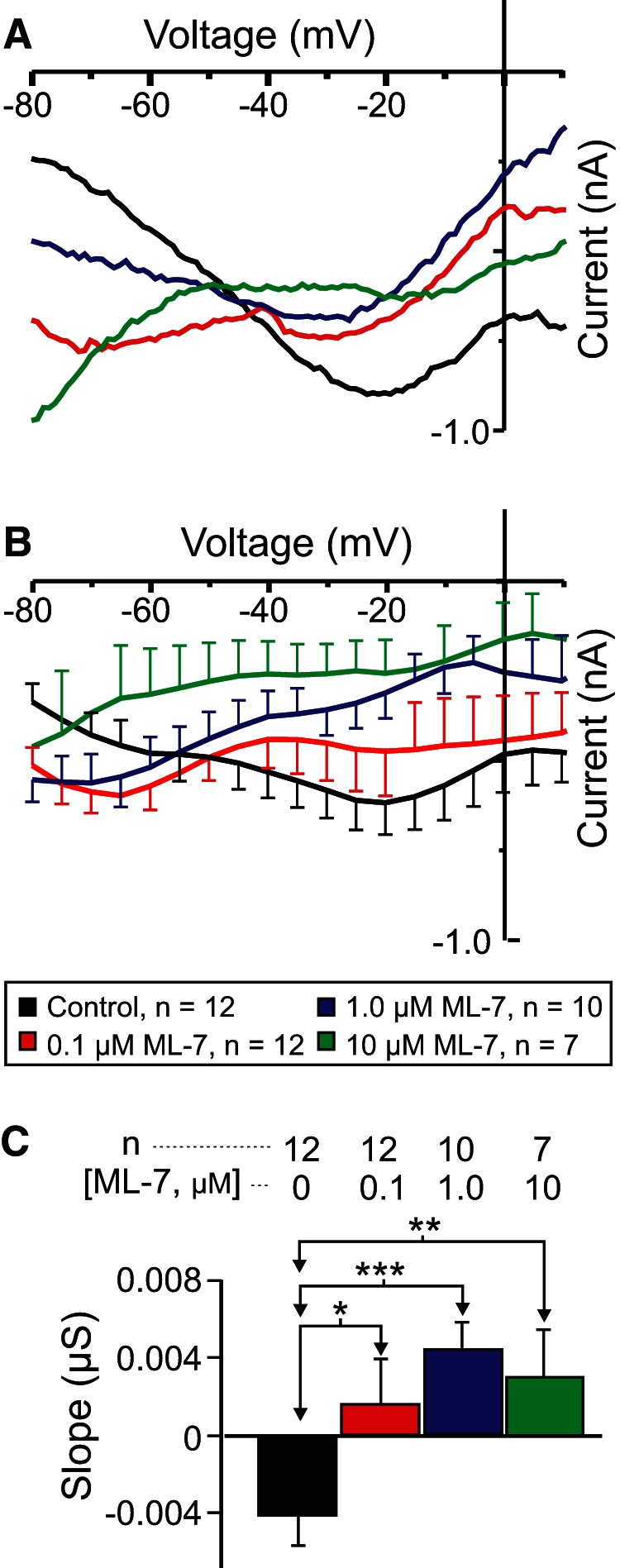
The MLCK inhibitor ML-7 reduces the voltage dependence of *I*_MI_ in LP neurons. ***A***, Representative *I–V* traces of proctolin-induced *I*_MI_ in the presence of various concentrations of ML-7. ***B***, Averaged *I–V* traces for all ML-7 experiments. ***C***, A one-way repeated-measures ANOVA showed that ML-7 increased proctolin-induced *I*_MI_ slope (*F*_(3,26)_ = 7.503, *p* = 8.92 × 10^−4ac^). Error bars indicate the SEM. Tukey’s test: *p < 0.05; **p < 0.01; ***p < 0.001.

MLCK is inhibited by W7 independently of calmodulin (Inagaki et al., 1986). It is involved in both muscarinic (So and Kim, 2003) and noradrenergic (Aromolaran et al., 2000) activation of sodium-permeable voltage-gated cationic currents in mammalian smooth muscle and bradykinin-induced reductions in manganese influx (and, presumably, intracellular calcium) in endothelial cells (Takahashi et al., 1997). However, most importantly, MLCK has been proposed to act downstream of CaSR signaling (Conigrave et al., 2007). For these reasons, we measured proctolin-induced *I*_MI_ in the presence of the specific MLCK inhibitor ML-7, which is known to be effective in other crustacean systems (Yamamoto et al., 1998; Chen et al., 2012). Our prediction was that, if MLCK mediates CaSR signaling, we should observe a dose-dependent increase in *I*_MI_ slope. As shown in Figure 7, *A* and *B*, ML-7 increased proctolin-induced *I*_MI_ slope in a dose-dependent manner. A one-way repeated-measures ANOVA showed that ML-7 significantly increased proctolin-induced *I*_MI_ slope (*F*_(3,26)_ = 7.503, *p* = 8.92 × 10^−4ac^; Fig. 7*C*, Table 3), which is consistent with our hypothesis that CaSR mediates *I*_MI_ voltage dependence. A *post hoc* Tukey’s test showed that this was significant at concentrations as low as 0.1 µm (*p* = 0.024). ML-7 also appeared to reduce proctolin-induced *I*_MI_ amplitude at high concentrations. A one-way repeated-measures ANOVA showed that ML-7 decreased proctolin-induced *I*_MI_ amplitude at −15 mV (*F*_(3,26)_ = 4.468, *p* = 0.012^ad^), with a *post hoc* Tukey’s test showing that this inhibition was significant only at concentrations of ≥1 µm (*p* = 0.035; data not shown).

### Preincubation in the endocytosis inhibitor dynasore prevents W7-induced reduction in voltage dependence

**Figure 8.**
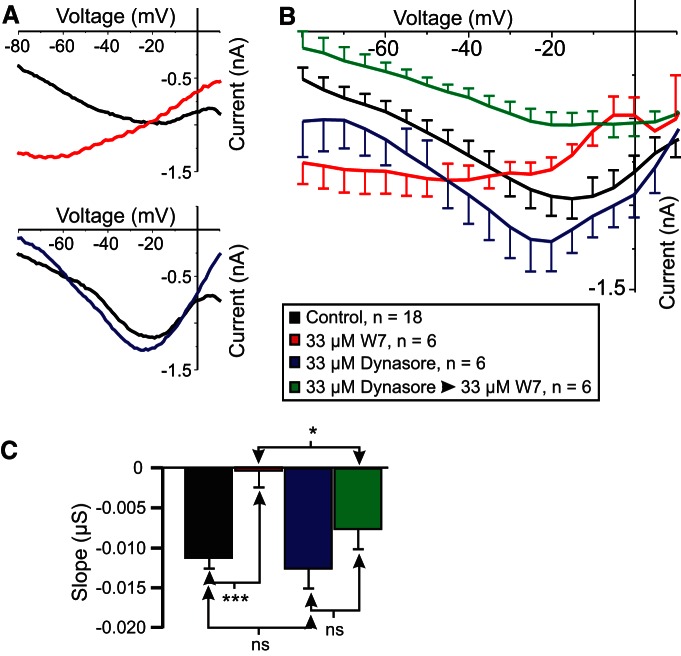
The endocytosis inhibitor dynasore prevents W7-induced increases in *I*_MI_ slope in LP neurons. CCAP-induced *I*_MI_ was measured before (black) and after exposure to the following: 33 µm W7 for 45-65 min (red); dynasore for 45-80 min (blue); 20 min in 33 µm dynasore, then 45-60 min in 33 µm dynasore plus 33 µm W7 (green). ***A***, Representative *I–V* curves of W7 effect (top) and dynasore effect (bottom). ***B***, Average *I–V* curves of all experiments. ***C***, Average slope of CCAP-induced *I*_MI_ from data in ***B*** and two-way ANOVA for the effects of factors dynasore and W7 on slope (W7: *F*_(1,32)_ = 14.934, *p* = 5.12 × 10^−4^; dynasore: *F*_(1,32)_ = 4.317, *p* = 0.046; Interaction: *F*_(1,32)_ = 2.109, *p* = 0.156^ae^). *Post hoc* Tukey’s comparisons on slope: (1) W7 only vs control (*p* < 0.001); (2) dynasore only vs control (*p* = 0.624); (3) dynasore only vs dynasore plus W7 (*p* = 0.129); and (4) W7 only vs W7 plus dynasore (*p* = 0.03). *Post hoc* Tukey’s test: *p < 0.05; ***p < 0.001.

If the loss of *I*_MI_ voltage dependence in W7 is due to the loss of CaSR signaling due to receptor endocytosis (Huang et al., 2010), then the blockade of endocytosis is expected to prevent W7-induced reductions in *I*_MI_ voltage dependence. To test this, we constructed a 2 × 2 experimental design using CCAP to induce *I*_MI_. *I*_MI_ was measured in the presence of a 33 μm concentration of the endocytosis inhibitor dynasore (cell-permeable dynamin inhibitor; IC_50_ for human Dyn1 and Dyn2, ∼15 µm; Macia et al., 2006) and an effective concentration of W7 (i.e., 33 μm; Fig. 2). A two-way ANOVA for factors W7 and dynasore showed a significant increase in CCAP-induced *I*_MI_ slope with W7 (as also observed with proctolin-induced *I*_MI_; Fig. 2*A*; W7: *F*_(1,32)_ = 14.934, *p* = 5.12 × 10^−4^; dynasore: *F*_(1,32)_ = 4.317, *p* = 0.046; interaction: *F*_(1,32)_ = 2.109, *p* = 0.156^ae^). The weak significance of the dynasore condition in this ANOVA originates entirely from the effect of dynasore in the presence of W7 (Fig. 8*B*,*C*, green vs red: *post hoc* Tukey’s test, *p* = 0.003; Table 3). CCAP-induced *I*_MI_ shows a markedly increased slope in the presence of 33 μm W7 alone (Fig. 8*A–C*, red) compared with control (black traces; *post hoc* Tukey’s test, *p* < 0.001; Table 3). However, in contrast to W7 alone, and consistent with our prediction, when cells were preincubated in 33 µm dynasore, and then exposed to 33 µm W7 in the presence of dynasore (Fig. 8*B*,*C*, green), no significant difference was observed when compared with dynasore alone (Fig. 8*B*,*C*, blue): *post hoc* Tukey’s test, *p* = 0.129 (Table 3). Note that incubation in dynasore alone (Fig 8*A–C*, blue) had no significant effect on *I*_MI_ slope compared with control (*p* = 0.624; Table 3). These results indicate that incubation in dynasore prevents W7-induced loss of voltage dependence of *I*_MI_. Although we did not expect a change in CCAP-induced amplitude at −15 mV, a two-way ANOVA for factors dynasore and W7 showed that W7, but not dynasore, produced significant changes in *I*_MI_ amplitude (W7: *F*_(1,32)_ = 8.738, *p* = 0.006; dynasore: *F*_(1,32)_ = 0.0001, *p* = 0.991; interaction: *F*_(1,32)_ = 1.472, *p* = 0.234^af^). However, a Tukey’s test showed that this effect arose only from a significant difference between dynasore and W7 preincubated with dynasore (*p* = 0.011), while the other treatments did not significantly contribute to this trend.

### The specific CaSR agonist R568 reduces *I*_MI_ voltage dependence

**Figure 9.**
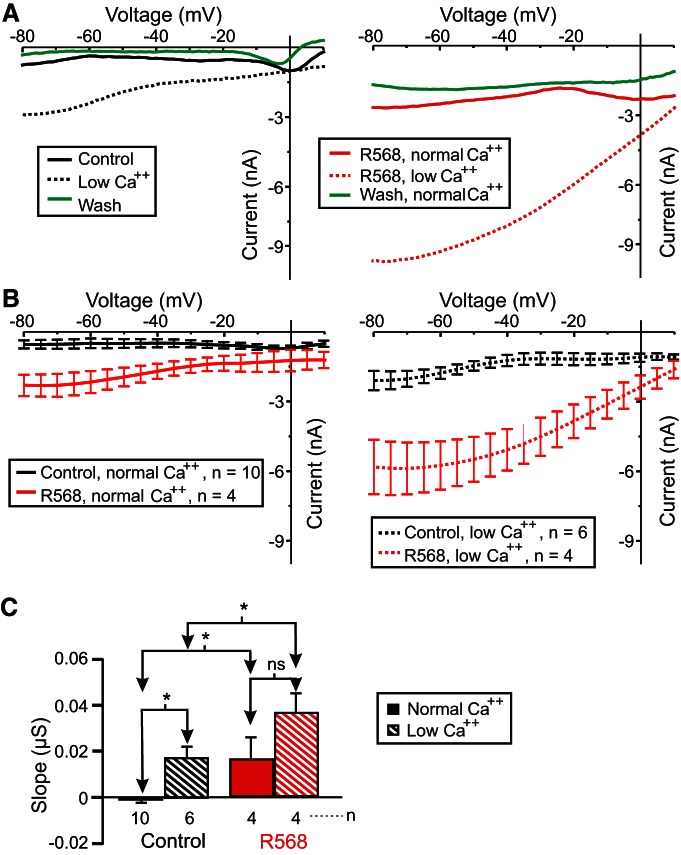
The CaSR agonist R568 increases proctolin-induced *I*_MI_ slope in LP neurons. Proctolin-induced *I*_MI_ was measured in the presence (red) or absence (black) of 10 µm CaSR agonist R568 in either 13 mm CaCl2 (solid) or 2 mm CaCl2 (striped). ***A***, Left, Representative *I–V* curves of proctolin-induced *I*_MI_ in 13 mm CaCl2 (black solid), 2 mm CaCl2 (black dotted), and 13 mm CaCl2 (green solid) after a 1 h wash. Right, Representative *I–V* curves of proctolin-induced *I*_MI_ in the presence of 10 µm CaSR agonist R568 in normal (13 mm) calcium (red solid), low (2 mm) calcium (red dotted), and then in normal calcium after 1 h wash from R568 (green solid). ***B***, Left, Averaged *I–V* traces of all proctolin-induced *I*_MI_ experiments in normal calcium in the presence (red solid) or absence (black solid) of 10 µm R568. Right, Averaged *I–V* traces of all proctolin-induced *I*_MI_ experiments in a low-calcium condition in the presence (red dotted) or absence (black dotted) of 10 µm R568. ***C***, Quantification of *I*_MI_ slope. Two-way ANOVA for factors R568 and calcium showing significant changes in proctolin-induced *I*_MI_ slope (calcium: *F*_(1,20)_ = 8.560, *p* = 0.008; R568: *F*_(1,20)_ = 9.295, *p* = 0.006; interaction: *F*_(1,20)_ = 0.0324, *p* = 0.859^ag^). Error bars indicate the SEM. Tukey’s test: *p < 0.05; ***p < 0.001.

Because we observed an increase in the proctolin-induced *I*_MI_ slope in the presence of the CaSR antagonist NPS-2143, we predicted that incubation in the specific CaSR agonist R568 should decrease or leave the slope unaltered in normal calcium, but should make the slope more negative (restore voltage dependence) in low-calcium conditions. In contrast to our expectations, 10 µm R568 increased both proctolin-induced *I*_MI_ slope and amplitude in both normal and low-calcium conditions (Fig. 9*A*,*B*). A two-way ANOVA for factors R568 and calcium showed significant changes in proctolin-induced *I*_MI_ slope (calcium: *F*_(1,20)_ = 8.560, *p* = 0.008; R568: *F*_(1,20)_ = 9.295, *p* = 0.006; interaction: *F*_(1,20)_ = 0.0324, *p* = 0.859^ag^; Fig. 9*C*). Instead of the slope decreasing as expected, a *post hoc* Tukey’s test showed that in a normal calcium condition, the proctolin-induced *I*_MI_ slope increased upon exposure to R568 instead (*p* = 0.047). Likewise, in a low-calcium condition, R568 increased proctolin-induced *I*_MI_ slope (*p* = 0.041; Fig. 9*C*, Table 3). These results are consistent with the hypothesis that CaSR modulates *I*_MI_ voltage dependence, but are inconsistent with the predicted direction of this modulation (see Discussion). In contrast with our expectation that *I*_MI_ amplitude should not be affected, a two-way ANOVA for factors R568 and calcium showed significant changes in proctolin-induced *I*_MI_ amplitude at −15 mV (calcium: *F*_(1,20)_ = 18.301, *p* = 3.67 × 10^−4^; R568: *F*_(1,20)_ = 23.447, *p* = 9.87 × 10^−5^; interaction: *F*_(1,20)_ = 5.962, *p* = 0.024^ah^). A *post hoc* Tukey test showed that the amplitude change was not significant in the normal calcium condition (*p* = 0.091) but was significant in the low-calcium condition (*p* < 0.001; data not shown).

## Discussion

The voltage dependence of ion channels is most commonly due to the properties of a voltage sensor embedded in the structure of the ion channels themselves. Exceptions to this mechanism are the voltage-dependent block of NMDA channels by magnesium (Ascher et al., 1988), a CaMKII-mediated phosphorylation of cGMP-activated channels in dogfish retinal bipolar cells (Shiells and Falk, 2001), calcineurin-mediated voltage dependence in salamander bipolar cells (Snellman and Nawy, 2002), and activation of G-proteins in mammalian smooth muscle (Zholos and Bolton, 1996). Here we report evidence that a calmodulin-sensitive pathway activated by extracellular calcium-sensing receptors may constitute a novel mechanism of voltage dependence of the neuromodulator-activated *I*_MI_ in crab STG neurons.

**Figure 10.**
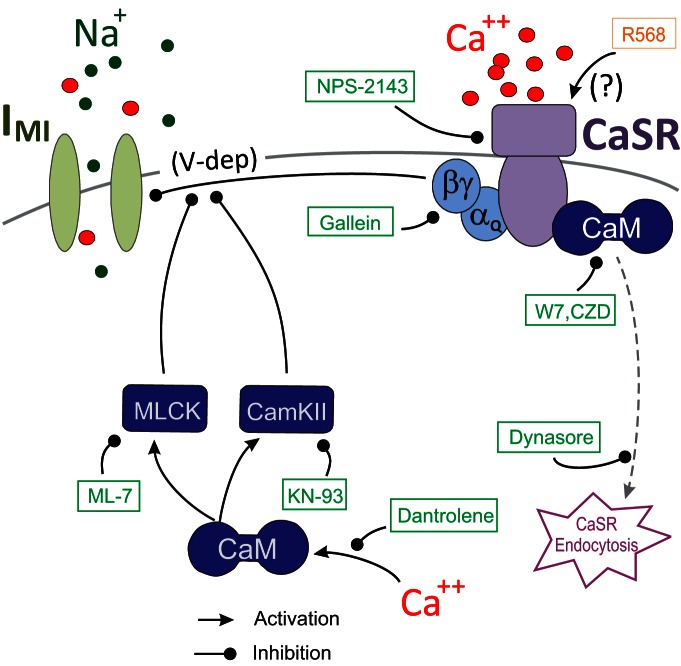
A model for CaSR-mediated regulation of *I*_MI_ voltage dependence. According to this model, *I*_MI_ channels are activated by a neuropeptide receptor using a pathway (not shown here) independent from the depicted voltage dependence pathway. The voltage dependence of *I*_MI_ is regulated by G-protein-coupled CaSRs. Calmodulin (CaM) stabilizes the receptor on the membrane, and inhibitors of calmodulin lead to CaSR endocytosis. Green boxes and blunt-ended lines show agents that inhibit the indicated paths. Arrows indicate activating pathways. Intracellular calcium release via ryanodine receptors is part of the source for calmodulin activation, and both CaMKII and MLCK inhibit voltage dependence.

Previous findings showed that extracellular calcium regulates the voltage dependence of *I*_MI_ in a manner analogous to the voltage-dependent magnesium block of NMDA channels (Golowasch and Marder, 1992b). Subsequently, the calmodulin inhibitor W7 was found to affect *I*_MI_ amplitude and perhaps voltage dependence (Swensen and Marder, 2000). Based on this, we predicted that activated calmodulin is directly or indirectly involved in the generation of *I*_MI_ voltage dependence (Fig. 10, summary diagram). We hypothesized that the influx of calcium regulates calmodulin activity. This would explain the reduction of voltage dependence in low-extracellular calcium conditions. This was supported by our observations that calmodulin inhibitors reduced proctolin-induced *I*_MI_ voltage dependence (Fig. 2). Also supporting this is the finding that the ryanodine receptor antagonist dantrolene similarly decreased *I*_MI_ voltage dependence (Fig. 3), presumably by reducing intracellular calcium release. More indirect support was provided by inhibitors of calmodulin-activated proteins KN-93 and ML-7 (Figs. 4, 7, 10), which are capable of reducing *I*_MI_ voltage dependence. Unexpectedly, we found that, although *I*_MI_ voltage dependence in the presence of normal calcium can be reduced by inhibitors of calmodulin, voltage dependence cannot be restored in low-calcium solutions by calmodulin activators (e.g., CALP1). These results led us to conclude that calmodulin is necessary but not sufficient to give rise to *I*_MI_ voltage dependence.

### A new hypothesis: the calcium-sensing receptor hypothesis

We propose that calcium-sensing receptors actively monitor extracellular calcium activity and regulate *I*_MI_ voltage dependence via a separate pathway than its neuromodulator-dependent activation pathway (Fig. 10). We are aware that this hypothesis does not represent a complete understanding of the mechanism of calcium-dependent voltage control of *I*_MI_, but identifies what we believe are major players in this system. We expect this hypothesis to constitute a framework for understanding our results and for a more detailed examination of these ideas and their implications in the future. A related mechanism has been proposed for the regulation of activation of leak currents in other systems (Lu et al., 2010). According to this hypothesis, when extracellular calcium is lowered, CaSR signaling to *I*_MI_ is reduced, and *I*_MI_ voltage dependence is consequently decreased. This is supported primarily by the finding that the CaSR antagonist NPS-2143 inhibits *I*_MI_ voltage dependence (Fig. 6). Further, consistent with existing views of CaSR function (Huang et al., 2010), we claim that calmodulin antagonist-induced loss of voltage dependence is due to CaSR endocytosis, which is supported by our observation that endocytosis inhibitors prevent W7-induced reduction in voltage dependence (Fig. 8). Although all our data were collected in LP cells, we predict that CaSR may play a similar role in modulating *I*_MI_ in all other neurons of the pyloric network since all display the peptide-induced current *I*_MI_ (Swensen and Marder, 2000), at least one additional identified neuron (the PD neuron) has been shown to lose voltage dependence in low-calcium conditions (Zhao et al., 2010), and Swensen and Marder (2000) showed that some additional unidentified neurons also show *I*_MI_ linearization in low-external calcium conditions.

Interestingly, the specific CaSR agonist R568 (Nemeth et al., 2001) did not restore *I*_MI_ voltage dependence in low-calcium conditions, as expected (Fig. 9). In the absence of a positive control for R568 in crustaceans, one possible explanation consistent with a model of CaSR-mediating voltage dependence would be homologous desensitization of CaSR (Gama and Breitwieser, 1998), with agonist-dependent phosphorylation and β-arrestin interactions as a possible mechanism (Pi et al., 2005; Mudò et al., 2009; Bandyopadhyay et al., 2010; Lu et al., 2010; Vysotskaya et al., 2014; Vizard et al., 2015), although CaSRs in other systems are noted for their resistance to desensitization (Grant et al., 2011; Breitwieser, 2013; Conigrave and Ward, 2013). According to our model, the inability of R568 to restore voltage dependence in low-calcium conditions suggests that strong stimulation of CaSR by R568 could lead to endocytosis of the receptor and loss of voltage dependence. Future studies should test the effects of R568 in low-calcium conditions on CaSR after the inhibition of endocytosis or of G-protein receptor kinases with the expectation that this should lead to the restoration of *I*_MI_ voltage dependence.

### Is CaSR signaling required for stable expression of neuromodulator-activated *I*_MI_?

We have observed that the desensitization of proctolin- and CCAP-induced *I*_MI_ is significantly faster in low-calcium compared with normal calcium conditions (Table 2). One possible explanation is that a minimum amount of CaSR signaling may be required to stabilize *I*_MI_ channels and their activation by neuromodulators. It is unclear, however, whether this would occur at the level of neuromodulator receptors, second messenger pathways, or the *I*_MI_ channels. In agreement with *I*_MI_ requiring activated CaSR signaling is the finding that the inhibition of MLCK, a suspected CaSR second messenger (Conigrave et al., 2007), reduced *I*_MI_ amplitude. However, NPS-2143, a CaSR antagonist, would then be expected to reduce *I*_MI_ activation, something we did not observe. Further, gallein, which is known to reduce plasma membrane expression of CaSR and phosphoinositide hydrolysis in response to CaSR agonists in other systems (Grant et al., 2011), should, in this case, reduce *I*_MI_ amplitude. We have in fact observed an inhibiting effect of gallein on *I*_MI_, but this was not statistically significant. Unfortunately, without a direct assay for CaSR, we cannot determine directly whether gallein is affecting CaSR receptor expression. An indirect means for assaying this in future studies would be to survey the ability of NPS-2143 to alter *I*_MI_ voltage dependence at different gallein concentrations.

### Is voltage dependence a target for meta-modulation?

The findings that voltage dependence may be modulated by a G-protein-coupled CaSR, together with the finding that the Gβγ inhibitor gallein reduced *I*_MI_ voltage dependence, implicating the G-protein βγ-subunit in the regulation of *I*_MI_ voltage dependence, suggests that voltage dependence itself could be a target of neuromodulation. Consistent with this is the observation of Zhao et al. (2010) that the negative linear slope region of the *I–V* curve of *I*_MI_ is all that is required to induce oscillatory activity in this system. This observation was used to claim that *I*_MI_ in these cells is a pacemaker current (Zhao et al., 2010). This indicates that oscillatory activity could be regulated by modulation of the voltage dependence, in addition to the activation levels, of a current. Voltage dependence has been shown to be a target of second messenger pathways before, albeit in a nonoscillatory system (Zholos and Bolton, 1996; Nawy, 2000; Shiells and Falk, 2001; Snellman and Nawy, 2002). Therefore, it would be interesting to examine the comodulation in this system in further detail to see whether voltage dependence itself could be the direct target of neuromodulation.

To conclude, the novel role for extracellular calcium reported here adds another layer of complexity to the role of calcium in the regulation of neuronal activity. Besides its ubiquitous role as intracellular second messenger of calcium, there is mounting evidence that calcium plays an increasingly important role as an extracellular ligand. Not only does calcium regulation play a vital role in parathyroid (Ho et al., 1995; McGehee et al., 1997; Pi et al., 2005), bone (Kameda et al., 1998), intestine (Cheng et al., 2002; Conigrave et al., 2007; Grant et al., 2011), and kidney function (Aida et al., 1995), but increasingly also in the modulation of neuronal activity (Mudò et al., 2009; Bandyopadhyay et al., 2010; Lu et al., 2010; Vysotskaya et al., 2014; Vizard et al., 2015).

### What is the physiological role of CaSR in the stomatogastric ganglion?

As Zhao et al. (2010) have suggested, the negative slope conductance region of *I*_MI_ controls the oscillatory character of these neurons. Thus, local extracellular calcium concentration changes could create a gradient of activity from tonic spiking at low concentrations, where *I*_MI_ is less voltage dependent, to the classic triphasic pyloric slow rhythm at normal calcium concentrations, in which negative slope conductance is present. Thus, the crucial question is: do extracellular calcium levels change significantly for CaSR activity to be affected? In contrast to vertebrates, in invertebrates extracellular calcium can vary dramatically during molting (Ziegler et al., 2000; Ahearn et al., 2004). On the other hand, in other systems, CaSR has been proposed to inhibit synaptic release when extracellular calcium levels are high through a presynaptic mechanism. In this mechanism, CaSR inhibits a nonselective cation current in normal calcium conditions, but when local extracellular calcium drops, disinhibition of this current compensates for the expected drop in synaptic release probability (Phillips et al., 2008; Jones and Smith, 2016). In mammals, local external calcium is known to vary significantly under different conditions, both normal and pathological, as follows: reductions of up to ∼30% are observed after 30 s of stimulation in cerebellum (Nicholson et al., 1978; Nicholson, 1980); reductions of >50% are observed during seizure activity in cerebral cortex (Heinemann et al., 1977); overall depletion occurs in hippocampus (Rusakov and Fine, 2003); and significant decreases occur in dorsal root ganglion (Galvan et al., 1979). Furthermore, in agreement with the activity of neurons being controlled by external calcium concentration, is the finding that CaSR is expressed in neurons in hippocampus (Mudò et al., 2009; Kim et al., 2014; Bai et al., 2015; Dong et al., 2015), cerebellum (Kapoor et al., 2008), cortex (Kapoor et al., 2008; Phillips et al., 2008; Vyleta and Smith, 2011), striatum after experimentally induced stroke (Noh et al., 2015), and sensory neurons (Vysotskaya et al., 2014). If similar changes in external calcium levels were demonstrated in the STG, then CaSRs could indeed regulate pyloric activity by modifying the level of *I*_MI_ voltage dependence.

Finally, it is possible that CaSR functions as a sensor of amino acid level fluctuations, as it is known to do in the mammalian intestine (Conigrave and Ward, 2013). This would add another level of modulation of the pyloric network that has not yet been explored.

## Synthesis

The decision was a result of the Reviewing Editor Alfonso Araque and the peer reviewers coming together and discussing their recommendations until a consensus was reached. A fact-based synthesis statement explaining their decision and outlining what is needed to prepare a revision is listed below. The following reviewers agreed to reveal their identity: Abdel EI Manira, Ronald Calabrese

Synthesis of the reviews

I concur with the reviewers' comments expressing the interest and quality of the study. And I also consider pertinent the concerns expressed. I believe that addressing those concerns, which is probably relatively easy, will definitely improve the manuscript.

Specific comments of the reviewers:

Reviewer 1.

This study describes a novel mechanism of voltage-dependent neuromodulation in stomatogastric ganglion neurons. This manuscript extends on previous studies related to voltage-dependent modulation of an inward current and provides convincing evidence that, unlike what was previously thought, the voltage-dependence involves activation of a calcium sensing receptor. The figures are clear and easy to follow and the manuscript is well written.

I have only a few minor comments/suggestions to be considered by the authors when revising this manuscript.

1) It is not clear which neurons were record in this study. Do all neurons in the STG exhibit this voltage-dependent neuromodulation? It would be helpful to clarify which neurons were recorded and if the effect studied is ubiquitous to all neurons.

2) I am curious to see what effect does the antagonist of the CaSR has on the rhythmic motor pattern of the stomatogastric ganglion. i) does it affect the pyloric rhythm? ii) Does it alter the magnitude of the effects of proctolin or CCAP? This information, if available, would be valuable to link this novel neuromodulatory mechanisms to network function.

Reviewer 2.

The authors present pharmacological analysis of an important current that is modulator activated. This current activated by a plethora of peptides is called IMI and is found in many neurons of the important model system, the crustacean stomatogastric nervous system (STN). It is a voltage dependent slow inward current mainly carried by Na+ and is characterized by a negative slope resistance in it IV plot. It is a relatively small current, derived as a difference current in voltage-clamp ramp protocols, but it is an important determinant of rhythmicity in the STN. From it first discovery by Freschi (1989), it has been enigmatic because its IV plot loses in negative slope resistance in low calcium saline and becomes a linear leak-like current. Until this study the current hypothesis concerning this linearization was that there was a voltage dependent Ca2+ block analogous to the Mg2+ block of NMDA channels. This view was complicated by the observation of Swensen and Marder (2000) that the calmodulin blocker W7 enhanced IMI in a voltage-dependent manner and concluded that a calmodulin-dependent pathway was likely involved in IMI activation. This study aims to show that the situation is much more complex; it purports to show that the Ca sensitivity of voltage dependence is mediated by CaSRs and involves calmodulin, MLCK, and CamKII.

This reviewer cannot critically evaluate the subtleties of the pharmacological characterization; I have little experience with the drugs used and cannot comment on their appropriateness or specificity. I therefore give the benefit of the doubt that the drugs work as advertised. Nevertheless the results are sufficiently complex and not without surprises/conflicts so that the whole story in not totally clear and certainly not definitive. On the other hand, there is a truly tremendous amount of experimental work here and the data is carefully analyzed and statistically tested. One also wishes that the difference currents were not so heterogeneous and squiggly looking but the current is a small one on a large leak background; I tend to trust that the experimenters know what they are measuring.

Two concerns reduce my enthusiasm for this work.

1. The results do not give a complete or definitive picture but seem rather to identify players and to require some hand-waving (CaSR agonist effects) to result in a model that is more a framework than a mechanistic understanding (Fig. 10).

2. Try as they might in Discussion the authors fail to convince me that the elaborate mechanism they suggest has physiological meaning: what are physiological conditions under which voltage sensitivity would be modulated by this mechanism and to what function?

Minor Concerns:

1. Line 21: "...and CaSR agonists and antagonists affected IMI voltage-dependence." seems to hide a lot of complexity especially in Abstract. Agonists have effects opposite prediction.

2. Line 325 and subsequent section; in the absence of a positive control for efficacy in this system the CALP1 results are not definitive.

3. Line 339 and subsequent section: Experiments that increase intracellular Ca2+ from stores in low external Ca2+ seems important here. Thapsigargin????

4. Lines 375-77: "These results (for dantrolene) are consistent with previous calmodulin inhibitor results, which suggest a role for intracellular calcium and calmodulin in the regulation of IMI voltage-dependence." Why is there no conclusion about activation when similar data for KN-93 mentions effects on both voltage dependence and activation.

5. Fig. 4A: There is no mention in the text of the green dashed line or is there reference to prolonged applications of KN-93.

6. Line 428-31: These two sentences are confusingly redundant.

7. Fig 9C: Displaced Y-axis labels.

8. Lines 565-72: This is an inappropriate personal communication. We cannot judge the likelihood of a CaSR critically based on such evidence.

7. Lines 583-86: There appear to be missing words.

8. Lines 627-29: Besides the ubiquitous role of calcium as an intracellular second messenger, there is mounting evidence that calcium plays an increasingly important role as an extracellular ligand
